# Balancing the effects of solar radiation pressure on the orbital elements of a spacecraft using Lorentz force

**DOI:** 10.1038/s41598-022-20166-y

**Published:** 2022-09-22

**Authors:** M. A. Yousef, M. I. El-Saftawy, A. Mostafa

**Affiliations:** 1 Department of Astronomy and Space Science, Faculty of Science, King Abdulaziz University, Jeddah, Kingdom of Saudi Arabia; 2 National Research Institute of Astronomy and Geophysics, Helwan, Cairo, Egypt; 3 Department of Mathematics, Faculty of Science, Ain Shams University, Cairo, Egypt

**Keywords:** Astronomy and astrophysics, Planetary science

## Abstract

In this work, orbits of Lorentz spacecrafts and satellites are investigated under the perturbation of solar radiation pressure. An attempt is made to control the perturbation of the solar radiation pressure using the effect of Lorentz force that affects an electrically charged spacecraft. The charge per unit mass is the controlling parameter in this process. The redial, transverse and normal components of the mentioned forces are constructed. The Lagrange planetary equations for perturbations in the Keplerian orbital elements are formulated. The formula describing the charge per unit mass, as function of the physical parameter of the problem as well as the orbital elements, were derived. The effects of the combined forces are analytically and numerically studied.

## Introduction

The orbital theory of the artificial bodies, either artificial satellites or spacecrafts, is one of the most essential science disciplines. This importance appears due to engaging its applications with human life such as communication, climate change, environmental science, and Earth science. Based on the purpose of the orbit, the orbits are designed differently. So, there are six Keplerian orbital elements of spacecraft that determine its orbit^1^. As the time goes with any involving outer perturbing force on the satellite, the orbital parameters are significantly affected.

To obtain desired orbits, the forces involved in any mathematical model must be solved. These forces involved consist of two parts. Firstly, major forces define the major terms of the orbits. Secondly, perturbing forces determine the deviations of the orbit from its main one. These deviations cause the artificial satellite to leave the planned orbit with time. The main objective of the orbital theory is to minimize the effects of the perturbing forces as much as possible.

In space, there are gravitational and nongravitational disturbances that can affect any satellite during traveling on its orbit. The gravitational disturbance engages with central attraction mass of the large body on the small body (satellite). On the other hand, the effects of nongravitational disturbance have a significant impact on the motion of the satellite. Based on the altitudes, the nongravitational disturbances influence the satellite on different ways. For example, in the low orbit altitudes the atmospheric drag as a nongravitational disturbance affects the satellite’s motion. For higher orbit altitudes, the interaction of the satellite’s surface with solar photons produces a force which is called SRP. The effects of the SRP on any satellite depends on many parameters such as distance to the Sun and satellite’s position with respect to Sun and Earth. Any modeling of the SRP depends on the accurate performance of the satellite orbit, the behavior of the satellite, and the geometric and physical properties of the satellite structures^2,3^.

Recently, the electromagnetic force of the Earth’s magnetic field on a charged object has been studied and used to control the motion of the artificial object. The effect of this force is called Lorentz force or LF^4^. This force is used to balance perturbations of the same order of LF within the range of possible charges that can be produced nowadays.

Obviously, the urgent need for orbits, whose space orientation and in turn coverage regions, remains fixed for somewhat extended intervals of time. Orbits satisfying these conditions are called frozen orbits^5–7^. The use of LF in orbit control is an interacting process between space dynamics and space technology which is responsible for designing the cells that produce the charge over the space craft. The literature is rich now with articles in this field, e.g.^8,9^.

In this paper, we will investigate the condition (or conditions) to control all orbital elements to avoid the effects of SRP as perturbing force using Lorentz acceleration. The LPE’s are formed for the combined forces using the charge per unit mass as a controller parameter. After that, the formed equations are solved to determine the required values of charge per unit mass that balance the effects of SRP.

In "Problem formulation" section explains both forces, SRP and LF, with presenting their components. In "Methodology" section, the Lagrange’s planetary equations (LPE) is formulated under both effects SRP and LF. After that the average perturbations, over an orbital revolution, are obtained. In "Balancing the SRP perturbation using LF" section calculates the controlling orbital elements. Some numerical applications are used to calculate the required values of charge per unit mass as in "Numerical application" section. Lastly, in "Conclusion and discussion" section provides a short summary of our results. The results show the applicability of using LF to balance SRP.

## Problem formulation

### Solar radiation pressure (SRP)

Let $T_{SRP}$, $R_{SRP}$, and $W_{SRP}$ be the transverse, redial, and normal components of the SRP, affected spacecraft with effective cross-sectional area, *A* and the mass *m*, respectively. These components, mathematically, can be written as^10–13^:

1.1 $$R_{SRP} = - \frac{{r_{0}^{2} }}{{r_{ \odot }^{2} }}R\left( \gamma \right)\left[ {\psi_{1} Cos \nu + \psi_{2} Sin\nu } \right],$$

1.2 $$T_{SRP} = - \frac{{r_{0}^{2} }}{{r_{ \odot }^{2} }}R\left( \gamma \right)\left[ { - \psi_{1} Sin\nu + \psi_{2} Cos \nu } \right],$$

1.3 $$W_{SRP} = - \frac{{r_{0}^{2} }}{{2r_{ \odot }^{2} }}R\left( \gamma \right)\psi_{3} ,$$

with,

$$\begin{aligned} \psi_{1} & = \frac{1}{4}\left[ \begin{gathered} \left( {1 + C} \right)\left( {1 + C_{ \odot } } \right){\text{Cos}}\left( {\theta - \omega - \Omega } \right) + \left( {1 - C} \right)\left( {1 + C_{ \odot } } \right){\text{Cos}}\left( {\theta + \omega - \Omega } \right) + \left( {1 - C} \right)\left( {1 - C_{ \odot } } \right){\text{Cos}}\left( {\theta - \omega + \Omega } \right) \hfill \\ + \left( {1 + C} \right)\left( {1 - C_{ \odot } } \right){\text{Cos}}\left( {\theta + \omega + \Omega } \right) + 2S{\text{S}}_{ \odot } {\text{Cos}} \left( {\theta - \omega } \right) - 2S{\text{S}}_{ \odot } {\text{Cos}}\left( {\theta + \omega } \right) \hfill \\ \end{gathered} \right], \\ \psi_{2} & = \frac{1}{4}\left[ \begin{gathered} \left( {1 + C} \right)\left( {1 + C_{ \odot } } \right){\text{Sin}}\left( {\theta - \omega - \Omega } \right) - \left( {1 - C} \right)\left( {1 + C_{ \odot } } \right){\text{Sin}}\left( {\theta + \omega - \Omega } \right) + \left( {1 - C} \right)\left( {1 - C_{ \odot } } \right){\text{Sin}}\left( {\theta - \omega + \Omega } \right) \hfill \\ - \left( {1 + C} \right)\left( {1 - C_{ \odot } } \right){\text{Sin}}\left( {\theta + \omega + \Omega } \right) + 2S{\text{S}}_{ \odot } {\text{Sin}}\left( {\theta - \omega } \right) + 2S{\text{S}}_{ \odot } {\text{Sin}}\left( {\theta + \omega } \right) \hfill \\ \end{gathered} \right], \\ \psi_{3} & = \left[ {2CS_{ \odot } {\text{Sin }}\theta - S\left( {1 + C_{ \odot } } \right) Sin\left( {\theta - \Omega } \right) + S\left( {1 - C_{ \odot } } \right){\text{Sin}}\left( {\theta + \Omega } \right)} \right], \\ \end{aligned}$$

and,

$$\begin{gathered} R\left( \gamma \right) = \frac{A}{m}\frac{{S_{0} }}{c}\left( {1 + \alpha } \right) Cos^{2} \gamma . \hfill \\ S = Sin i,\,\,\,C = Cos i,\,\,\,C_{ \odot } = Cos \varepsilon \,\,{\text{and}}\,\,S_{ \odot } = Sin \varepsilon . \hfill \\ \end{gathered}$$.

$S_{0}$, $\theta$, $\varepsilon$ and *c* are the solar constant, the true longitude of the Sun, the obliquity of ecliptic and the speed of light respectively. $r_{0}$ and $r_{\Theta }$ are respectively the distances of the Earth and the spacecraft from the Sun. In addition, $\gamma$ and $\alpha$ are the falling angle of solar ray with the normal to the surface and the reflection coefficient of the spacecraft’s surface respectively.

The classical orbital parameters, used, are a, e, *i*, $\omega$, $\Omega$ and $\nu$ are the semi-major axis, eccentricity, inclination, argument of preside, argument of ascending node and true anomaly respectively.

### Lorentz force (LF)

Paul and Frueh^14^, defined the LF as a charged space object (satellite) which is moving through the magnetic field of the Earth. This charge produces instability in the currents of plasma electron, ion, photoelectric, secondary, and other different currents that the satellite usually exposed to.

The force affecting a charged spacecraft with charge per unit mass, *q*, moving in a magnetic field with magnetic dipole moment, *B*, rotating with rotational speed, $\vartheta$, can be written in redial, $R_{LF}$, transverse, $T_{LF}$, and normal, $W_{LF}$, components respectively as^15,16^:

2.1 $$T_{LF} = B_{1} \left( \frac{a}{r} \right)^{2} Sin 2v + B_{2} \left( \frac{a}{r} \right)^{2} Cos 2v + B_{3} \left( \frac{a}{r} \right)^{3} Sin v,$$

2.2 $$R_{LF} = \left[ {A_{1} \left( \frac{a}{r} \right)^{2} + A_{2} \left( \frac{a}{r} \right)^{4} + A_{3} \left( \frac{a}{r} \right)^{2} Cos 2v + A_{4} \left( \frac{a}{r} \right)^{2} Sin 2v } \right],$$

2.3 $$\begin{aligned} W_{LF} & = C_{0}^{c} \left( \frac{a}{r} \right)^{3} + \left[ {C_{1}^{c} \left( \frac{a}{r} \right)^{4} + C_{2}^{c} \left( \frac{a}{r} \right)^{2} } \right] Cos v + C_{3}^{c} \left( \frac{a}{r} \right)^{3} Cos 2v + \left[ {C_{1}^{s} \left( \frac{a}{r} \right)^{4} + C_{2}^{s} \left( \frac{a}{r} \right)^{2} } \right] Sin v \\ & \,\,\,\, + C_{3}^{s} \left( \frac{a}{r} \right)^{3} Sin 2v, \\ \end{aligned}$$

where,

$$B_{1} = \frac{{BqS^{2} \vartheta }}{{a^{2} }} Cos 2 \omega$$, $$B_{2} = \frac{{BqS^{2} \vartheta }}{{a^{2} }} Sin 2 \omega$$, $$B_{3} = \frac{Bq e C}{{a^{3} }}\sqrt {\frac{\mu }{p}}$$,

$$A_{1} = \frac{Bq\vartheta }{{a^{2} }} \left( {1 - \frac{1}{2}S^{2} } \right)$$, $$A_{2} = - \frac{{Bq C \sqrt {\mu p} }}{{a^{4} }}$$, $$A_{3} = \frac{{Bq\vartheta S^{2} }}{{2 a^{2} }} Cos 2 \omega$$, $$A_{4} = - \frac{{Bq\vartheta S^{2} }}{{2 a^{2} }} Sin 2 \omega$$,

$$C_{0}^{c} = \frac{B S q e}{{2 a^{3} }}\sqrt {\frac{\mu }{p}} Sin \omega$$, $$C_{1}^{c} = - \frac{{2 B S q \sqrt {\mu p} }}{{a^{4} }} Sin \omega$$, $$C_{2}^{c} = \frac{2 B S q C \vartheta }{{a^{2} }} Sin \omega$$, $$C_{3}^{c} = - C_{0}^{c}$$,

$$C_{1}^{s} = - \frac{{2 B S q \sqrt {\mu p} }}{{a^{4} }} Cos \omega$$, $$C_{2}^{s} = \frac{2 B S q C \vartheta }{{a^{2} }} Cos \omega$$, $$C_{3}^{s} = - \frac{B S q e}{{2 a^{3} }}\sqrt {\frac{\mu }{p}} Cos \omega$$.

## Methodology

### Lagrange’s planetary equations (LPE)

Let $n$, *h* and $r$ be the mean motion, specific angular momentum and polar redial distance from the center of mass of the planet, respectively. LPE can be used to calculate the perturbation on the spacecrafts orbit. Many textbooks can be used to describe and formulate LPE^17^, and^18^.

LPE’s can be written as:

3.1 $$\dot{a} = \frac{{2a^{2} }}{h}\left[ {T + e\left( {T Cos v + R Sin v} \right)} \right],$$

3.2 $$\dot{e} = \frac{{\sqrt {1 - e^{2} } }}{n a}\left[ {R Sin v + T \left( {Cos v + Cos E} \right)} \right],$$

3.3 $$\dot{i} = \frac{W}{h} r Cos\left( {\omega + v} \right),$$

3.4 $$\dot{\Omega } = \frac{W}{h Sin I} r Sin\left( {\omega + v} \right),$$

3.5 $$\dot{\omega } = \frac{h}{\mu e}\left[ { - R Cos v + T\left( { Sin v + \frac{1}{{\sqrt {1 - e^{2} } }} Sin E} \right)} \right] - \dot{\Omega } Cos I.$$

where *E* and *µ* are the eccentric anomaly and gravitational parameter of the planet respectively.

#### Perturbation due to SRP

To compute the perturbation due to SRP on the orbital elements, we must apply Eqs. (1.1)–(1.3) into Eqs. (3.1)–(3.5). After computing the required derivatives, we get:

4.1 $$\dot{a}_{SRP} = - \frac{{2a^{2} }}{h}\frac{{r_{0}^{2} }}{{r_{ \odot }^{2} }}R\left( \gamma \right)\left[ { - \psi_{1} Sin v + \psi_{2} Cos v + e \psi_{2} } \right],$$

4.2 $$\dot{e}_{SRP} = - \frac{{\sqrt {1 - e^{2} } }}{n a}\frac{{r_{0}^{2} }}{{r_{ \odot }^{2} }}R\left( \gamma \right)\left[ {\psi_{2} - \psi_{1} Sin\nu Cos E + \psi_{2} Cos \nu Cos E} \right],$$

4.3 $$\dot{i}_{SRP} = - \frac{{r_{0}^{2} }}{{2r_{ \odot }^{2} }}R\left( \gamma \right) \frac{{\psi_{3} }}{h} r Cos\left( {\omega + v} \right),$$

4.4 $$\dot{\Omega }_{SRP} = - \frac{{r_{0}^{2} }}{{2r_{ \odot }^{2} }}R\left( \gamma \right)\frac{{\psi_{3} }}{h S}\left( \frac{r}{a} \right) Sin\left( {\omega + v} \right),$$

4.5 $$\begin{aligned} \dot{\omega }_{SRP} & = \frac{h}{\mu e}\frac{{r_{0}^{2} }}{{r_{ \odot }^{2} }}R\left( \gamma \right)\left[ {\left( {\psi_{1} Cos \nu + \psi_{2} Sin\nu } \right) Cos v - \left( { - \psi_{1} Sin\nu + \psi_{2} Cos \nu } \right)\left( { Sin v + \frac{1}{{\sqrt {1 - e^{2} } }} Sin E} \right)} \right] \\ & \,\,\,\, - C\dot{\Omega }_{SRP} . \\ \end{aligned}$$

#### Perturbation due to LF

The perturbation due to LF on the orbital elements can be computed by substituting Eqs. (2.1)–(2.3) into Eqs. (3.1)–(3.5). After computing the required derivatives with simple mathematical manipulating, we get:

5.1 $$\dot{a}_{LF} = \frac{{2a^{2} }}{h}\left[ \begin{gathered} eA_{2} \left( \frac{a}{r} \right)^{4} Sin v + B_{3} \left( \frac{a}{r} \right)^{3} Sin v + \frac{e}{2}\left( {B_{1} + 2A_{1} - A_{3} + A_{4} } \right)\left( \frac{a}{r} \right)^{2} Sin v \hfill \\ + \frac{{eB_{3} }}{2}\left( \frac{a}{r} \right)^{3} Sin 2 v + B_{1} \left( \frac{a}{r} \right)^{2} Sin 2v + \frac{e}{2}\left( {B_{1} + A_{3} + A_{4} } \right)\left( \frac{a}{r} \right)^{2} Sin 3v \hfill \\ - \frac{{eB_{2} }}{2}\left( \frac{a}{r} \right)^{2} Cos v + B_{2} \left( \frac{a}{r} \right)^{2} Cos 2v + \frac{{eB_{2} }}{2}\left( \frac{a}{r} \right)^{2} Cos 3v \hfill \\ \end{gathered} \right],$$

5.2 $$\dot{e}_{LF} = \frac{{\sqrt {1 - e^{2} } }}{n a}\left[ \begin{gathered} A_{2} \left( \frac{a}{r} \right)^{4} Sin v + \frac{{B_{3} }}{2}\left( \frac{a}{r} \right)^{3} Sin 2v + \left( {\frac{{A_{3} }}{2} + \frac{{B_{1} }}{2}} \right)\left( \frac{a}{r} \right)^{2} Sin 3v + \left( {A_{1} - \frac{{A_{3} }}{2} + \frac{{B_{1} }}{2}} \right)\left( \frac{a}{r} \right)^{2} Sin v \hfill \\ + \left( {\frac{{B_{2} }}{2} - \frac{{A_{4} }}{2}} \right)\left( \frac{a}{r} \right)^{2} Cos 3v + \left( {\frac{{A_{4} }}{2} + \frac{{B_{2} }}{2}} \right)\left( \frac{a}{r} \right)^{2} Cos v \hfill \\ + B_{1} \left( \frac{a}{r} \right)^{2} Sin 2v Cos E + B_{2} \left( \frac{a}{r} \right)^{2} Cos 2v Cos E + B_{3} \left( \frac{a}{r} \right)^{3} Sin v Cos E \hfill \\ \end{gathered} \right],$$

5.3 $$\dot{i}_{LF} = \left\{ \begin{gathered} \left[ \begin{gathered} \frac{{C_{3}^{c} }}{2} \left( \frac{a}{r} \right)^{2} Cos 3v + \frac{1}{2}\left( {C_{1}^{c} \left( \frac{a}{r} \right)^{3} + C_{2}^{c} \left( \frac{a}{r} \right)} \right) Cos 2v \hfill \\ + \left( {C_{0}^{c} + \frac{{C_{3}^{c} }}{2}} \right)\left( \frac{a}{r} \right)^{2} Cosv + \frac{1}{2}\left( {C_{1}^{c} \left( \frac{a}{r} \right)^{3} + C_{2}^{c} \left( \frac{a}{r} \right)} \right) \hfill \\ + \frac{{C_{3}^{s} }}{2} \left( \frac{a}{r} \right)^{2} Sin 3v + \frac{1}{2}\left( {C_{1}^{s} \left( \frac{a}{r} \right)^{3} + C_{2}^{s} \left( \frac{a}{r} \right)} \right) Sin 2v + \frac{{C_{3}^{s} }}{2} \left( \frac{a}{r} \right)^{2} Sin v \hfill \\ \end{gathered} \right]Cos\omega \hfill \\ - \left[ \begin{gathered} \frac{{C_{3}^{c} }}{2} \left( \frac{a}{r} \right)^{2} Sin 3v + \frac{1}{2}\left( {C_{1}^{c} \left( \frac{a}{r} \right)^{3} + C_{2}^{c} \left( \frac{a}{r} \right)} \right) Sin 2v + \left( {C_{0}^{c} - \frac{{C_{3}^{c} }}{2} } \right)\left( \frac{a}{r} \right)^{2} Sin v \hfill \\ - \frac{{C_{3}^{s} }}{2} \left( \frac{a}{r} \right)^{2} Cos 3v - \frac{1}{2}\left( {C_{1}^{s} \left( \frac{a}{r} \right)^{3} + C_{2}^{s} \left( \frac{a}{r} \right)} \right)Cos 2v \hfill \\ + \frac{{C_{3}^{s} }}{2} \left( \frac{a}{r} \right)^{2} Cosv + \frac{1}{2}\left( {C_{1}^{s} \left( \frac{a}{r} \right)^{3} + C_{2}^{s} \left( \frac{a}{r} \right)} \right) \hfill \\ \end{gathered} \right]Sin \omega \hfill \\ \end{gathered} \right\}$$

5.4 $$\dot{\Omega }_{LF} = \frac{{W_{LF} }}{h Sin I} r Sin\left( {\omega + v} \right) = \frac{a}{h S}\left\{ \begin{gathered} \left[ \begin{gathered} - \frac{{C_{3}^{s} }}{2} \left( \frac{a}{r} \right)^{2} Cos 3v - \frac{1}{2}\left( {C_{1}^{s} \left( \frac{a}{r} \right)^{3} + C_{2}^{s} \left( \frac{a}{r} \right)} \right) Cos 2v \hfill \\ + \frac{{C_{3}^{s} }}{2} \left( \frac{a}{r} \right)^{2} Cos v + \frac{1}{2}\left( {C_{1}^{s} \left( \frac{a}{r} \right)^{3} + C_{2}^{s} \left( \frac{a}{r} \right)} \right) + \frac{{C_{3}^{c} }}{2} \left( \frac{a}{r} \right)^{2} Sin 3v \hfill \\ + \frac{1}{2}\left( {C_{1}^{c} \left( \frac{a}{r} \right)^{3} + \frac{{C_{2}^{c} }}{2}\left( \frac{a}{r} \right)} \right)Sin 2v + \left( {C_{0}^{c} - \frac{{C_{3}^{c} }}{2} } \right)\left( \frac{a}{r} \right)^{2} Sin v \hfill \\ \end{gathered} \right] Cos \omega \hfill \\ + \left[ \begin{gathered} \frac{{C_{3}^{c} }}{2} \left( \frac{a}{r} \right)^{2} Cos 3v + \frac{1}{2}\left( {C_{1}^{c} \left( \frac{a}{r} \right)^{3} + C_{2}^{c} \left( \frac{a}{r} \right)} \right)Cos 2v \hfill \\ + \left( {C_{0}^{c} + \frac{{C_{3}^{c} }}{2} } \right)\left( \frac{a}{r} \right)^{2} Cos v + \frac{1}{2}\left( {C_{1}^{c} \left( \frac{a}{r} \right)^{3} + C_{2}^{c} \left( \frac{a}{r} \right)} \right) + \frac{{C_{3}^{s} }}{2} \left( \frac{a}{r} \right)^{2} Sin 3v \hfill \\ + \frac{1}{2}\left( {C_{1}^{s} \left( \frac{a}{r} \right)^{3} + C_{2}^{s} \left( \frac{a}{r} \right)} \right) Sin 2v + \frac{{C_{3}^{s} }}{2} \left( \frac{a}{r} \right)^{2} Sin v \hfill \\ \end{gathered} \right]Sin \omega \hfill \\ \end{gathered} \right\},$$

5.5 $$\dot{\omega }_{LF} = - C\dot{\Omega }_{LF} + \frac{h}{\mu e}\left\{ \begin{gathered} - A_{2} \left( \frac{a}{r} \right)^{4} Cos v - B_{3} \left( \frac{a}{r} \right)^{3} Cos 2v \hfill \\ - \left( {\frac{{A_{3} }}{2} + \frac{{B_{1} }}{2}} \right)\left( \frac{a}{r} \right)^{2} Cos 3v + \left( {\frac{{B_{1} }}{2} - A_{1} - \frac{{A_{3} }}{2}} \right)\left( \frac{a}{r} \right)^{2} Cos v + B_{3} \left( \frac{a}{r} \right)^{3} \hfill \\ + \left( {\frac{{B_{2} }}{2} - \frac{{A_{4} }}{2}} \right)\left( \frac{a}{r} \right)^{2} Sin 3v - \left( {\frac{{A_{4} }}{2} + \frac{{B_{2} }}{2}} \right)\left( \frac{a}{r} \right)^{2} Sin v + \frac{{B_{1} }}{{\sqrt {1 - e^{2} } }}\left( \frac{a}{r} \right)^{2} Sin 2v Sin E \hfill \\ + \frac{{B_{2} }}{{\sqrt {1 - e^{2} } }}\left( \frac{a}{r} \right)^{2} Cos 2v Sin E + \frac{{B_{3} }}{{\sqrt {1 - e^{2} } }}\left( \frac{a}{r} \right)^{3} Sin v Sin E \hfill \\ \end{gathered} \right\}.$$

### Average orbital perturbations

In the next subsections we will calculate the averages of the perturbation due to SRP and LF over the complete period of the true anomaly $v$.

The average of the function *f(x)* over a complete period for the variable *x* is defined as:

6 $$f\left( x \right)_{x} = \frac{1}{2 \pi } \mathop \smallint \limits_{0}^{2 \pi } f\left( x \right)dx.$$

#### Average perturbation due to SRP

Now, apply the averaging definition for Eqs. (4.1)–(4.5). After calculating the required integrations, we get:

7.1 $$\left\langle {\dot{a}_{SRP} } \right\rangle_{v} = - \frac{{2a^{2} e}}{h}\frac{{r_{0}^{2} }}{{r_{ \odot }^{2} }}R\left( \gamma \right) \Psi_{2} ,$$

7.2 $$\left\langle {\dot{e}_{SRP} } \right\rangle_{\nu } = - \frac{{\sqrt {1 - e^{2} } }}{n a}\frac{{r_{0}^{2} }}{{r_{ \odot }^{2} }}R\left( \gamma \right)\left[ { \psi_{2} + \frac{{e^{4} - 3e^{2} + \left( {3e^{2} - 2} \right)\sqrt {1 - e^{2} } + 2}}{{e^{2} \left( {1 - e^{2} } \right)}}} \right],$$

7.3 $$\left\langle {\dot{i}_{SRP} } \right\rangle_{\nu } = - \frac{{r_{0}^{2} }}{{2r_{ \odot }^{2} }}R\left( \gamma \right) \frac{{\psi_{3} a }}{h} F\left( e \right) Cos \omega ,$$

7.4 $$\left\langle {\dot{\Omega }_{SRP} } \right\rangle_{\nu } = - \frac{{r_{0}^{2} }}{{2r_{ \odot }^{2} }}R\left( \gamma \right)\frac{{a \psi_{3} }}{h S} F\left( e \right) Sin \omega ,$$

7.5 $$\left\langle {\dot{\omega }_{SRP} } \right\rangle_{\nu } = - \frac{{r_{0}^{2} }}{{r_{ \odot }^{2} }}R\left( \gamma \right)\left\{ {\frac{{h\psi_{1} }}{{\mu e^{3} }}\left[ {\left( {\sqrt {1 - e^{2} } - 1 - e^{2} } \right)} \right] - \frac{{a \psi_{3} }}{h S} F\left( e \right) Sin \omega C} \right\},$$

where,

$$F\left( e \right) = \frac{{2 \left( {1 - e^{2} } \right)^{2} - \left( {e^{4} - 2 e^{2} + 2} \right)\sqrt {1 - e^{2} } }}{{e^{2} \left( {1 - e^{2} } \right)}}.$$

#### Average perturbation due to LF

Also, applying the averaging definition for Eqs. (5.1)–(5.5). After calculating the required integrations, we get:

8.1 $$\left\langle {\dot{a}_{LF} } \right\rangle_{v} = - \frac{{B q S^{2} e^{2} \vartheta }}{{2 h \left( {1 - e^{2} } \right)^{2} }} Sin 2 \omega ,$$

8.2 $$\left\langle {\dot{e}_{LF} } \right\rangle_{v} = - \frac{{B q \vartheta S^{2} e}}{{4 a^{3} n \left( {1 - e^{2} } \right)^{\frac{3}{2}} }} Sin 2 \omega ,$$

8.3 $$\left\langle {\dot{i}_{LF} } \right\rangle_{v} = \frac{{Be^{2} q S {\text{Sin}}2\omega }}{{2a^{3} \left( { - 1 + e^{2} } \right)^{3} }},$$

8.4 $$\left\langle {\dot{\Omega }_{LF} } \right\rangle_{v} = Bq\left[ {\frac{C \vartheta }{{p\sqrt {p\mu } }} - \frac{{ - \left( {2 + 3e^{2} } \right) + e^{2} {\text{ Cos}} 2\omega }}{{2p^{3} }}} \right],$$

8.5 $$\left\langle {\dot{\omega }_{LF} } \right\rangle_{v} = \frac{{{\text{Bq}}}}{{8p^{3} \sqrt {p\mu } }}\left\{ \begin{gathered} - 4C\left[ {2\sqrt {p\mu } + 2Cp^{2} \vartheta + e^{2} \sqrt {p\mu } \left( {3 - 2Cos\omega } \right)} \right] + aC\left( {12 - 5e^{2} - 7e^{4} } \right)\sqrt {\frac{\mu }{p}} \hfill \\ + 4C\left( {4 + 3e^{2} } \right)\sqrt {p\mu } + 4p^{2} \vartheta \left[ { - 2 + S^{2} \left( {1 + Cos\omega } \right)} \right] \hfill \\ \end{gathered} \right\}.$$

With, *p* is the semi-parameter of the orbit.

## Balancing the SRP perturbation using LF

To balance the effects of SRP perturbations on the Keplerian orbital elements, we need to determine the magnitude and type of the charge per unit mass that can balance such perturbation. After the LPE’s are formed for both forces, they are combined and solved using the charge per unit mass $\left( q \right)$, as controlling parameter. Then the desired values of $q$ are determined to balance such perturbation for each orbital element.

Let $q_{a}$, $q_{e}$, $q_{i}$, $q_{{\Omega }}$, and $q_{\omega }$ be the required charge per unit mass to balancing the variations in semi-major axis (a), eccentricity (e), inclination (*i*) argument of ascending node (${\Omega }$) and argument of preside ($\omega$) respectively. Using Eqs. (7.1)–(7.5) and (8.1)–(8.5), we get:

9.1 $$\begin{aligned} & \left\langle {\dot{a}_{SRP} } \right\rangle_{v} + \left\langle {\dot{a}_{LF} } \right\rangle_{v} = 0 \\ & \,\,\,\,\, - \frac{{2a^{2} e}}{h}\frac{{r_{0}^{2} }}{{r_{ \odot }^{2} }}R\left( \gamma \right) \psi_{2} - \frac{{B q S^{2} e^{2} \vartheta }}{{2 h \left( {1 - e^{2} } \right)^{2} }} Sin 2 \omega = 0 \\ q_{a} & = - \frac{{4 a^{2} \left( {1 - e^{2} } \right)^{2} { } R\left( \gamma \right) r_{0}^{2} \psi_{2} }}{{B e S^{2} \vartheta r_{ \odot }^{2} }} {\text{Csc }}2\omega \\ \end{aligned}$$

9.2 $$\begin{gathered} \left\langle {\dot{e}_{SRP} } \right\rangle_{v} + \left\langle {\dot{e}_{LF} } \right\rangle_{v} = 0 \hfill \\ \frac{{\sqrt {1 - e^{2} } }}{n a}\frac{{r_{0}^{2} }}{{r_{ \odot }^{2} }}R\left( \gamma \right)\left[ { \psi_{2} + \frac{{e^{4} - 3e^{2} + \left( {3e^{2} - 2} \right)\sqrt {1 - e^{2} } + 2}}{{e^{2} \left( {1 - e^{2} } \right)}}} \right] + \frac{{B q \vartheta S^{2} e}}{{4 a^{3} n \left( {1 - e^{2} } \right)^{\frac{3}{2}} }} Sin 2 \omega = 0 \hfill \\ q_{e} = - \frac{{4a^{2} \left( {1 - e^{2} } \right){ }R\left( \gamma \right) r_{0}^{2} }}{{B S^{2} \vartheta e^{3} r_{ \odot }^{2} }}\left[ {2 - 3e^{2} + e^{4} + \left( {3e^{2} - 2} \right)\sqrt {1 - e^{2} } + e^{2} \left( {1 - e^{2} } \right)\psi_{2} } \right]{\text{Csc}} 2\omega . \hfill \\ \end{gathered}$$

9.3 $$\begin{aligned} & \left\langle {\dot{i}_{SRP} } \right\rangle_{v} + \left\langle {\dot{i}_{LF} } \right\rangle_{v} = 0 \\ & \,\,\,\,\, - \frac{{r_{0}^{2} }}{{2r_{ \odot }^{2} }}R\left( \gamma \right) \frac{{\psi_{3} a }}{h} F\left( e \right) Cos \omega + \frac{{Be^{2} q S {\text{Sin}}2\omega }}{{2a^{3} \left( { - 1 + e^{2} } \right)^{3} }} = 0 \\ q_{i} & = - \frac{{a^{4} r_{0}^{2} R\left( \gamma \right) \psi_{3} F_{1} \left( e \right){ }}}{{r_{ \odot }^{2} B S h}} {\text{Cot}}\left[ \omega \right], \\ \end{aligned}$$

where,

$$F_{1} \left( e \right) = \frac{{\left( {1 - e^{2} } \right)^{3} }}{{ e^{2} }}F\left( e \right) = \frac{{\left( {1 - e^{2} } \right)^{2} \left[ {2 \left( {1 - e^{2} } \right)^{2} - \left( {e^{4} - 2 e^{2} + 2} \right)\sqrt {1 - e^{2} } } \right]}}{{e^{4} }}.$$

9.4 $$\begin{aligned} & \left\langle {{\dot{\Omega }}_{SRP} } \right\rangle_{v} + \left\langle {{\dot{\Omega }}_{LF} } \right\rangle_{v} = 0 \\ & \,\,\,\,\, - \frac{{r_{0}^{2} }}{{2r_{ \odot }^{2} }}R\left( \gamma \right)\frac{{a \psi_{3} }}{h S} F\left( e \right) Sin \omega + Bq\left[ {\frac{C \vartheta }{{p\sqrt {p\mu } }} - \frac{{ - \left( {2 + 3e^{2} } \right) + e^{2} {\text{ Cos}} 2\omega }}{{2p^{3} }}} \right] = 0 \\ q_{{\Omega }} & = \frac{{r_{0}^{2} R\left( \gamma \right) a p^{3} \psi_{3} F\left( e \right) Sin \omega }}{{r_{ \odot }^{2} S B \left[ {2 C \vartheta p^{2} + h \left( {2 + 3e^{2} - e^{2} {\text{ Cos}} 2\omega } \right) } \right]}}. \\ \end{aligned}$$

9.5 $$\begin{aligned} & \left\langle {\dot{\omega }_{SRP} } \right\rangle_{v} + \left\langle {\dot{\omega }_{LF} } \right\rangle_{v} = 0 \\ & \,\,\,\,\, - \frac{{r_{0}^{2} }}{{r_{ \odot }^{2} }}R\left( \gamma \right)\left\{ {\frac{{h\psi_{1} }}{{\mu e^{3} }}\left( {\sqrt {1 - e^{2} } - 1 - e^{2} } \right) - \frac{{a \psi_{3} }}{h S} F\left( e \right) Sin \omega C} \right\} \\ & \,\,\,\,\, + \frac{{{\text{Bq}}}}{{8p^{3} \sqrt {p\mu } }}\left\{ \begin{gathered} - 4C\left[ {2\sqrt {p\mu } + 2Cp^{2} \vartheta + e^{2} \sqrt {p\mu } \left( {3 - 2Cos\omega } \right)} \right] \hfill \\ + aC\left( {12 - 5e^{2} - 7e^{4} } \right)\sqrt {\frac{\mu }{p}} + 4C\left( {4 + 3e^{2} } \right)\sqrt {p\mu } + 4p^{2} \vartheta \left[ { - 2 + S^{2} \left( {1 + Cos\omega } \right)} \right] \hfill \\ \end{gathered} \right\} = 0 \\ q_{\omega } & = \frac{{8 p^{3} R\left( \gamma \right) r_{0}^{2} \left\{ {{\Gamma }_{1} - a C \psi_{3} F\left( e \right) Sin \omega } \right\}}}{{B S r_{ \odot }^{2} \left[ {{\Gamma }_{2} - {\Gamma }_{3} - 8 p^{2} \vartheta + 4 p^{2} \vartheta S^{2} \left( {1 + {\text{Cos }}\omega } \right)} \right]}}, \\ \end{aligned}$$

where,

$$\begin{gathered} {\Gamma }_{1} = \frac{{h^{2} S \psi_{1} }}{{\mu e^{3} }}\left( {\sqrt {1 - e^{2} } - 1 - e^{2} } \right), \hfill \\ {\Gamma }_{2} = 4h c\left[ {4 + 3e^{2} \left] { + a\sqrt {\frac{\mu }{p}} c} \right[12 - 5e^{2} - 7e^{4} } \right], \hfill \\ {\Gamma }_{3} = 4c\left[ {2c{ }p^{2} \vartheta + 2h + e^{2} h\left( {3 - 2{\text{Cos}}\left[ \omega \right]} \right)} \right]. \hfill \\ \end{gathered}$$

Equations (9.1)–(9.5) are the required relation needs to calculate the desired charge per unit mass to balance the effects of SRP on the spacecraft’s orbital elements according to required element and to the purpose of the balance. It is to be noted that elements can be balanced individually, or an optimization process can be made to get minimum possible perturbations to all elements at the same time. Near the discontinuity points, numerical investigation must be done using one of the numerical integrations with small step size.

The work deals with the secular perturbations only as they are the accumulative and the most effective forces on the satellite’s orbit. This has been processed by averaging over the fast angle of the orbit. This ensures a longer lifetime for the sat which is the main concern of the work. However, if there is a need for a local balance for a specific position the values of the charge per unit mass should be calculated without averaging.

The balance in both cases is guaranteed for each element by the fulfillment of the corresponding one of the conditions (9.1)-(9.5) with average or without average respectively.

The model is valid for regions with no shadow. It is known that there is no analytical treatment for shadow region yet.

## Numerical application

In this section, we will examine the charge per unit mass for different altitude satellites. We use the SEASAT satellite as low Earth orbit, LAGEOS satellite as mid Earth orbit and geostationary satellite as high Earth orbit.

The parameters for the planet Earth present in Table 1.

**Table 1:** The physical parameters for the Earth^19^.

B (T-Km^3^)	µ (km^3^/s^2^)	$\vartheta$(hr^-1^)	*ε* (^o^)	$S_{o} \left( {\frac{g}{{s^{3} }}} \right)$
8X10^6^	398,601	π/12	23.439	$1.3615 \times 10^{6}$

Calculations are done at θ = 0, for SEASAT, LAGEOS and Geostationary orbits display in Table 2.

**Table 2:** The parameters of the satellite’s in different altitudes^20^.

Satellite	a (Km)	e	i (^o^)	A/m (cm^2^ g^-1^)
SEASAT	7100	0.00209	108	0.2
LAGEOS	12,270	0.004456	109.8	0.007
Geostationary	42,160	0.00091	7.0	0.1

The dimensions used in the following calculation and figures are km, gram and second for distance, mass and time respectively while the charge per unit mass, *q*, are coulombs/gram.

## Conclusion and Discussion

From Eqs. (9.1)–(9.5) and the Figs. 1, 2, 3, 4, 5, 6, 7, 8, 9, 10, 11, 12, 13, 14, 15, we can summary our conclusions as follows:

- Because both SRP and LF perturbations depend on the charge per unit mass and area per unit mass, we can calculate the charge per unit area to cancel the mass of the spacecraft from equations.
- From Eq. (9.1), the function $q_{a}$ is discontinuous at $\left( {1 - Cos 2 i} \right) Sin 2 \omega$ which leads to $\omega = \left\{ {0 , \pm \frac{\pi }{2}} \right\}$ and $i = \left\{ {0 , \pm \pi } \right\}$. That means when the spacecraft is at the line of nods or normal to it or in equatorial orbit.
- The charge per unit mass is directly proportion with the square of the semi-major axis.
- The change of the *q* values from low to intermediate orbits is mainly due to the dependance on the different negative powers of the eccentricity (for semi-circular orbits *e* <  < *1*) while the change of the semi-major axis is within the same order of magnitude.
- For the geostationary orbits, the effective factors are the increases of *a* (squared) to 42,160 km. Also, the geostationary orbits have very small values of inclination, this increases the values of *q*’s which depend on (sin *i*) with different negative powers. The combined results of these two factors increase dramatically the value of the required charge to balance the under-study perturbations specially for the elements that inverse square with *Sin i*.

**Figure 1:**
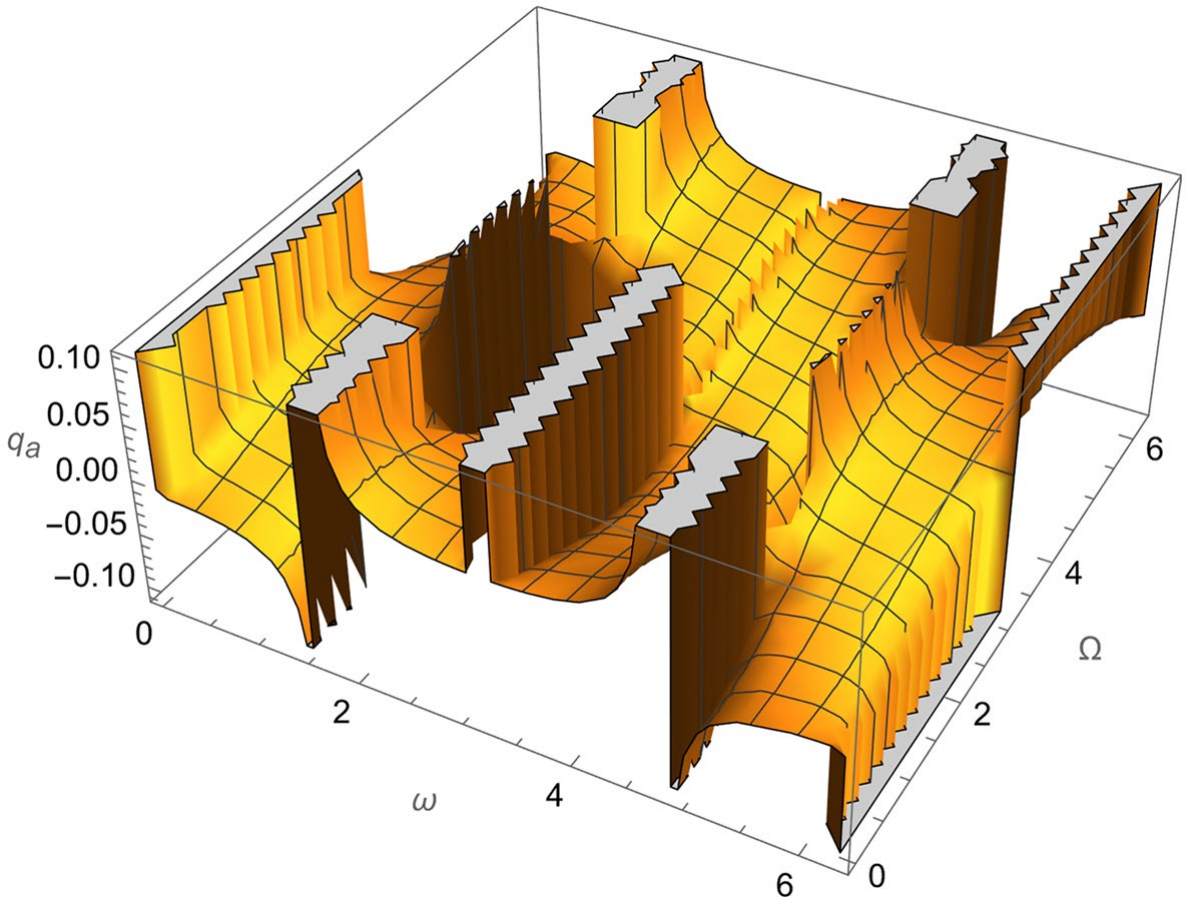
The variation of $\omega$ and $\Omega$ with $q_{a}$ for SESAT type satellite as a low Earth orbit.

**Figure 2:**
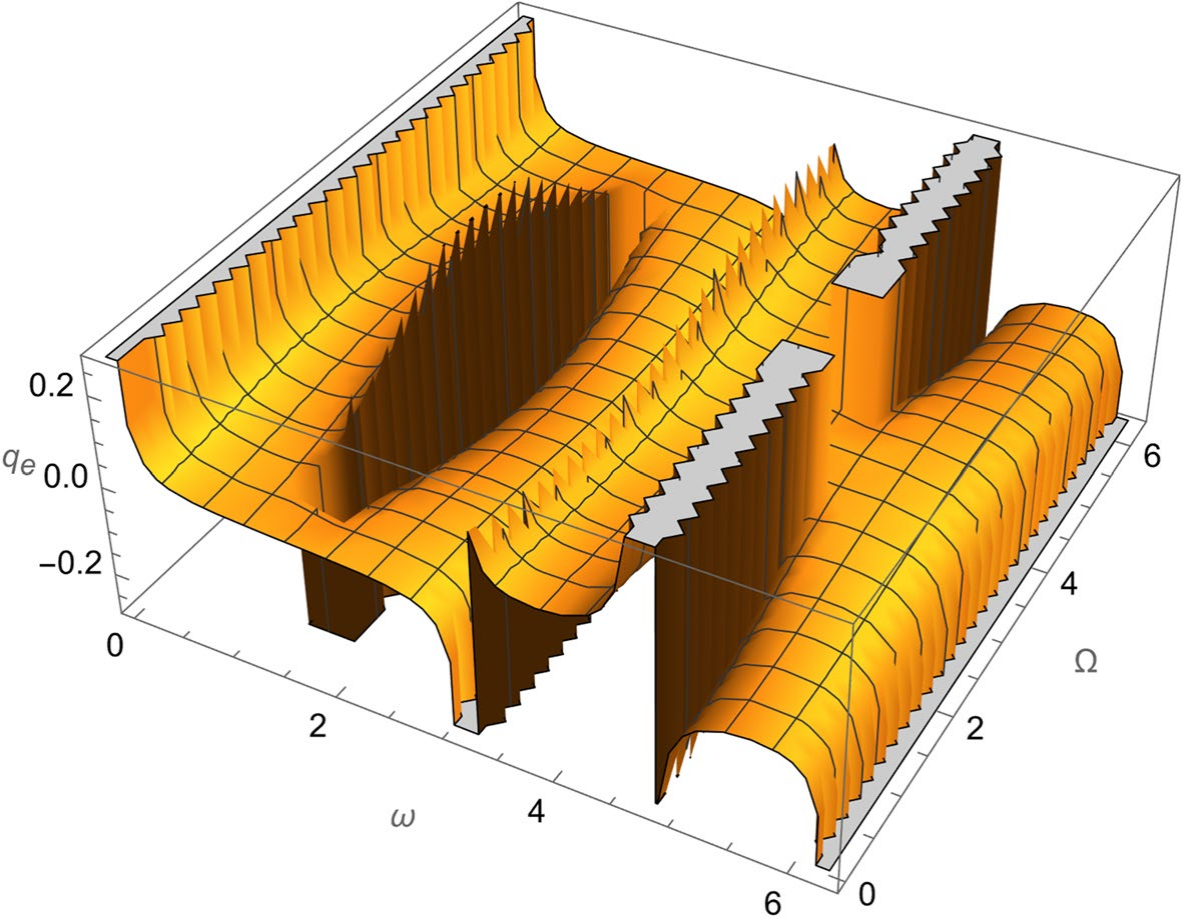
The variation of $\omega$ and $\Omega$ with $q_{e}$ for SESAT type satellite as a low Earth orbit.

**Figure 3:**
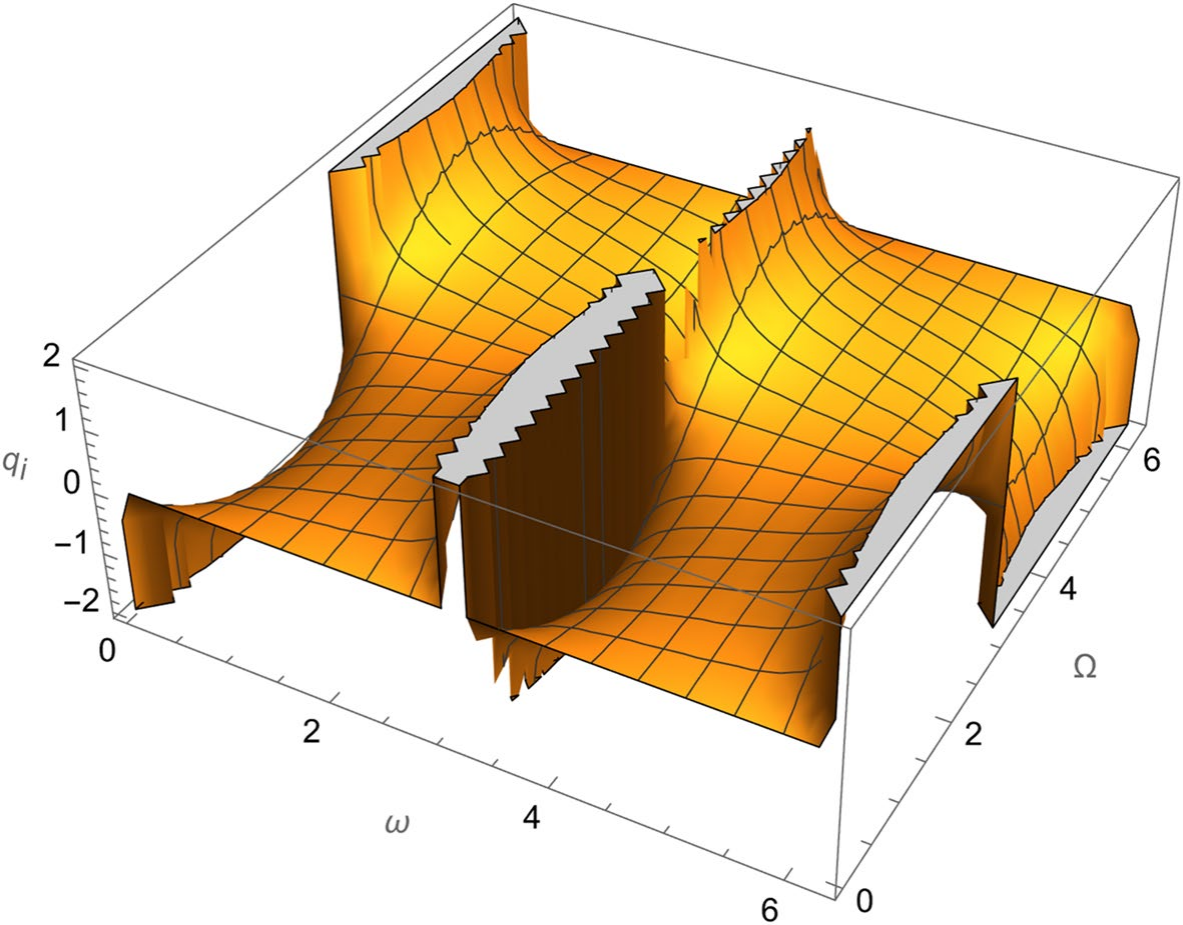
The variation of $\omega$ and $\Omega$ with $q_{i}$ for SESAT type satellite as a low Earth orbit.

**Figure 4:**
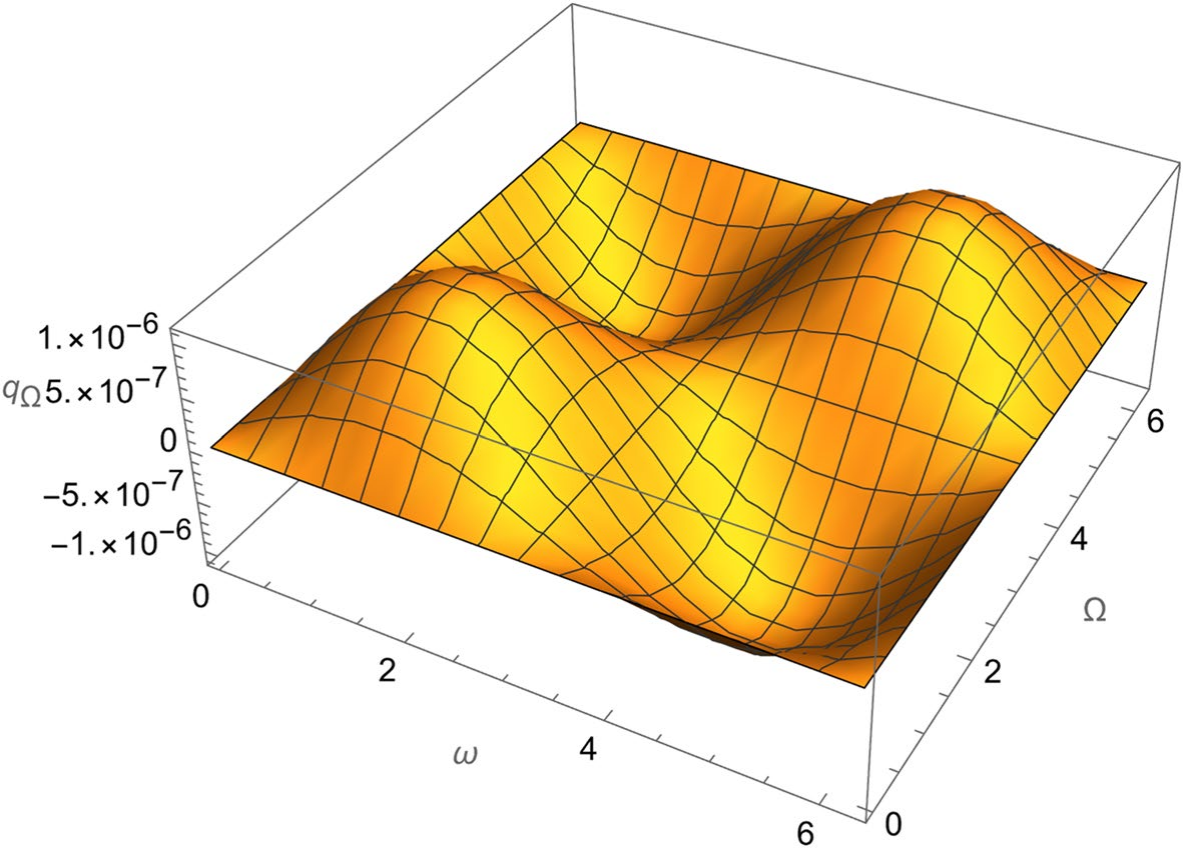
The variation of $\omega$ and $\Omega$ with $q_{\Omega }$ for SESAT type satellite as a low Earth orbit.

**Figure 5:**
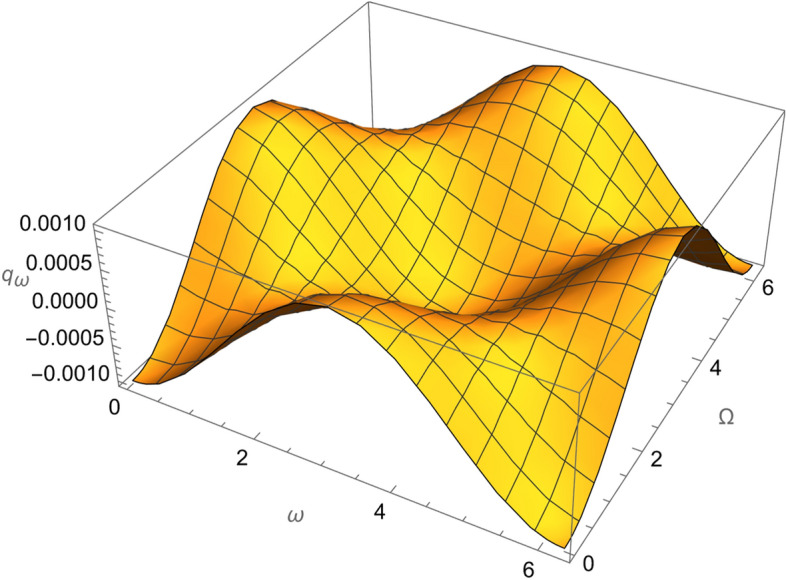
The variation of $\omega$ and $\Omega$ with $q_{\omega }$ for SESAT type satellite as a low Earth orbit.

**Figure 6:**
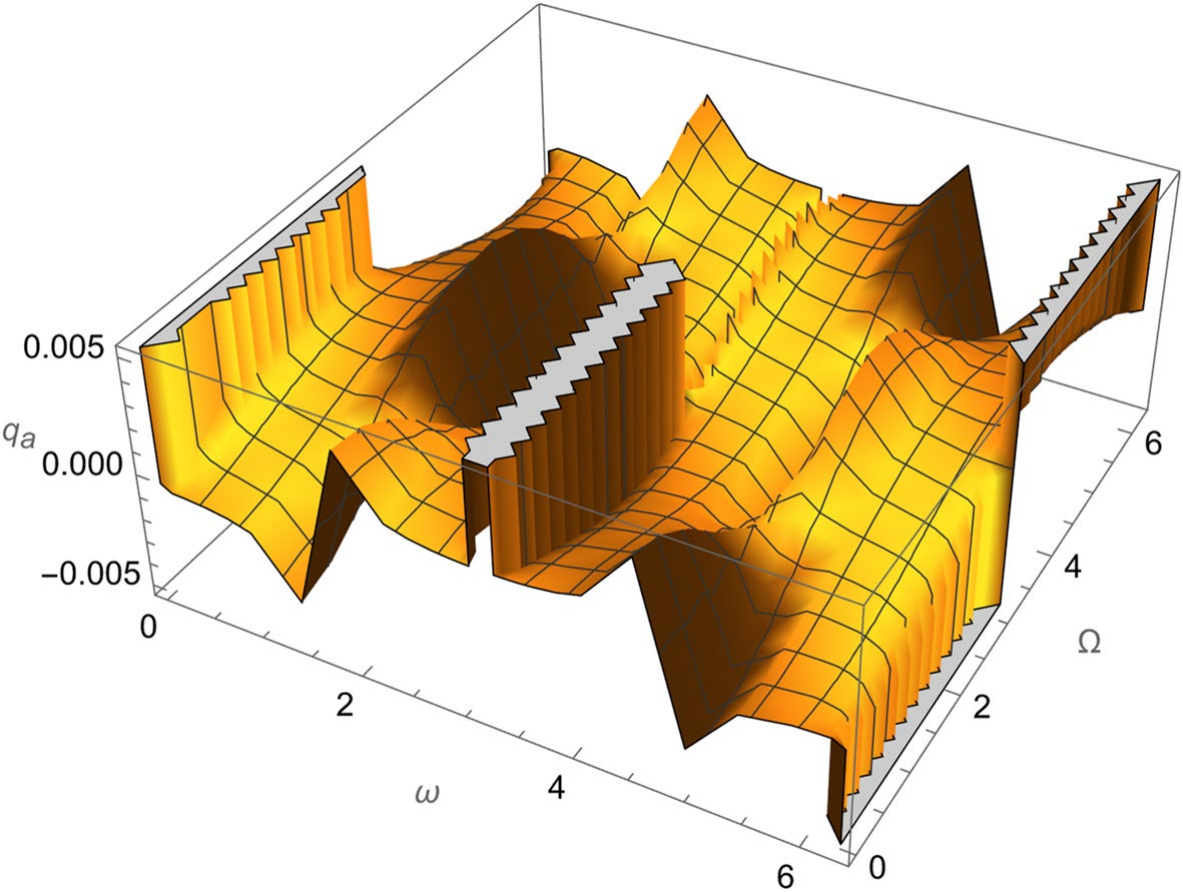
The variation of $\omega$ and $\Omega$ with $q_{a}$ for LAGEOS type satellite as a mid-Earth orbit.

**Figure 7:**
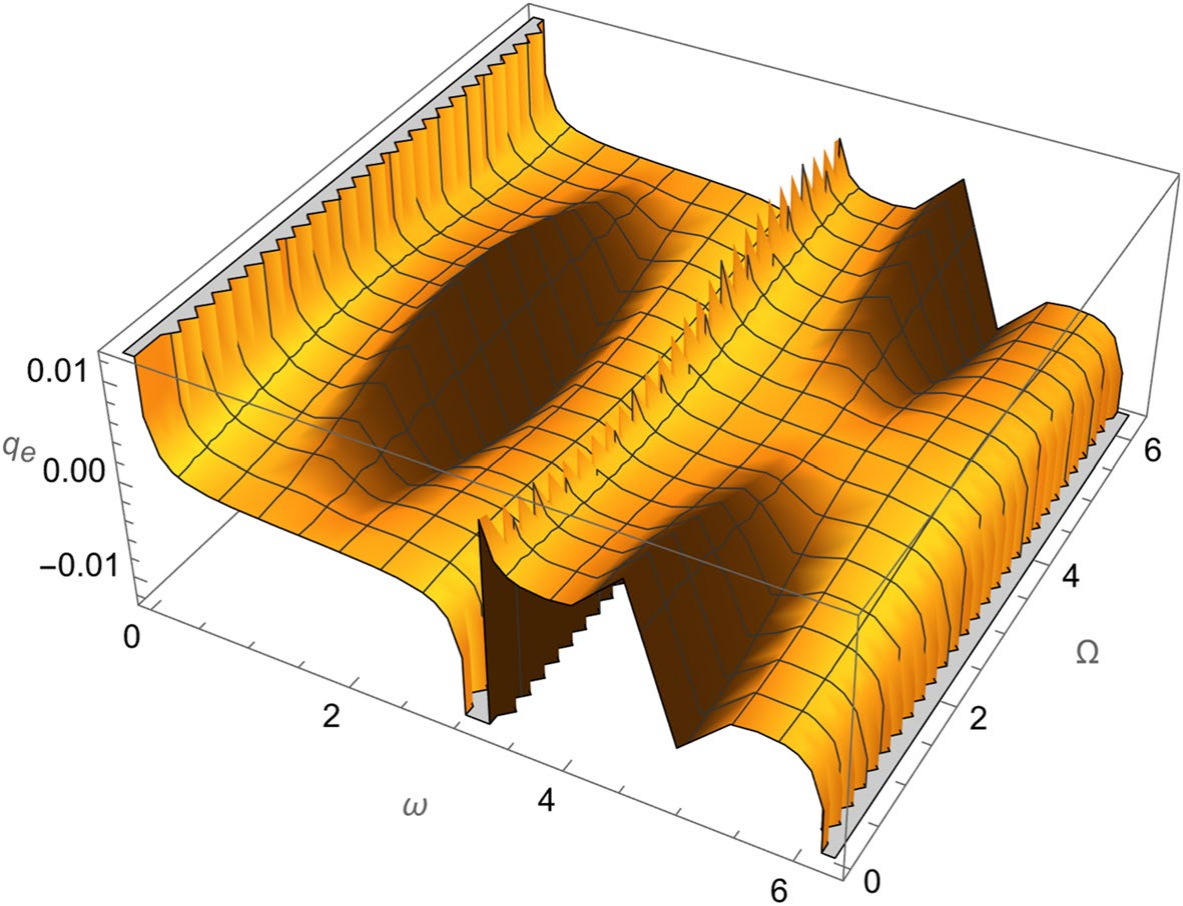
The variation of $\omega$ and $\Omega$ with $q_{e}$ for LAGEOS type satellite as a mid-Earth orbit.

**Figure 8:**
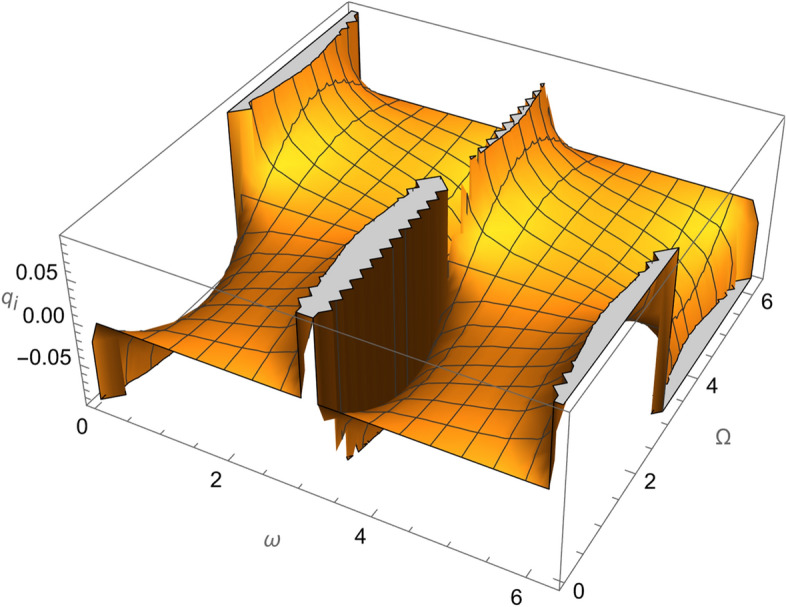
The variation of $\omega$ and $\Omega$ with $q_{i}$ for LAGEOS type satellite as a mid-Earth orbit.

**Figure 9:**
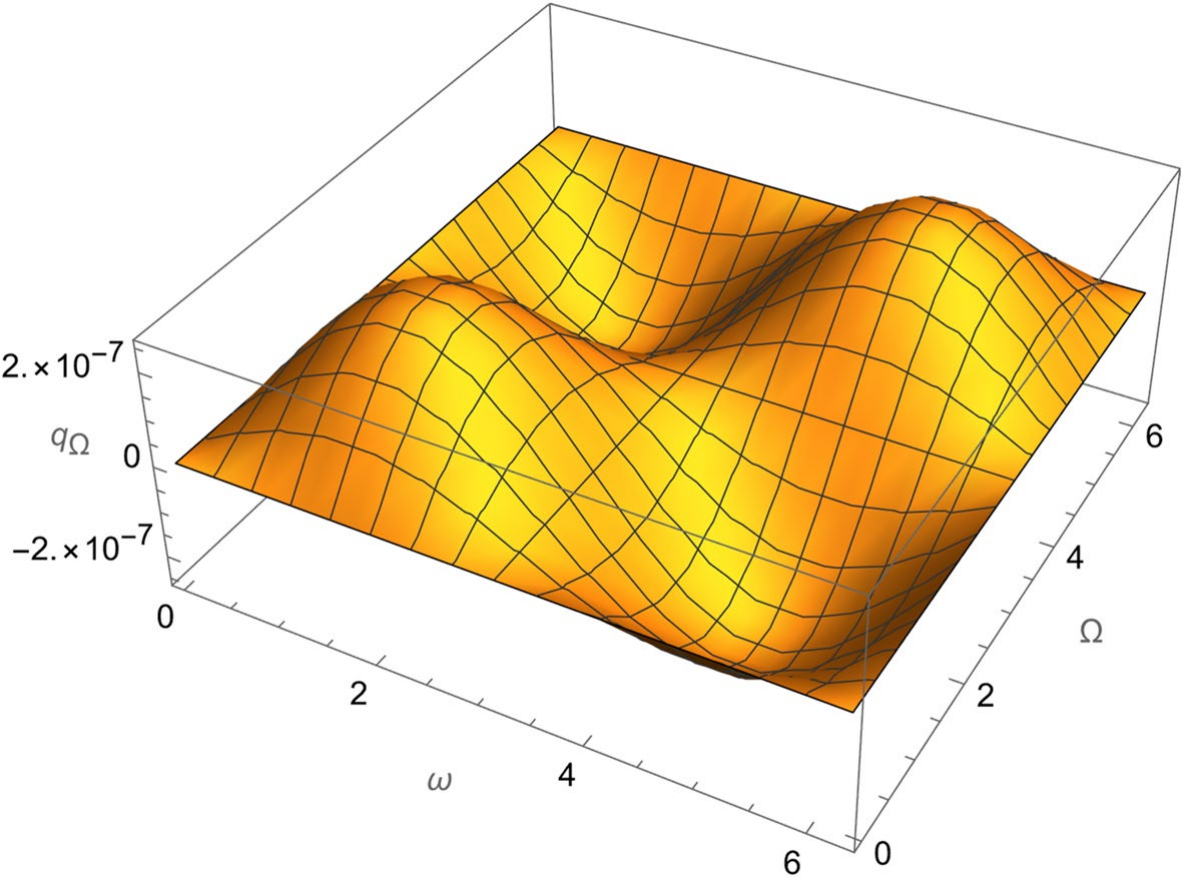
The variation of $\omega$ and $\Omega$ with $q_{\Omega }$ for LAGEOS type satellite as a mid-Earth orbit.

**Figure 10:**
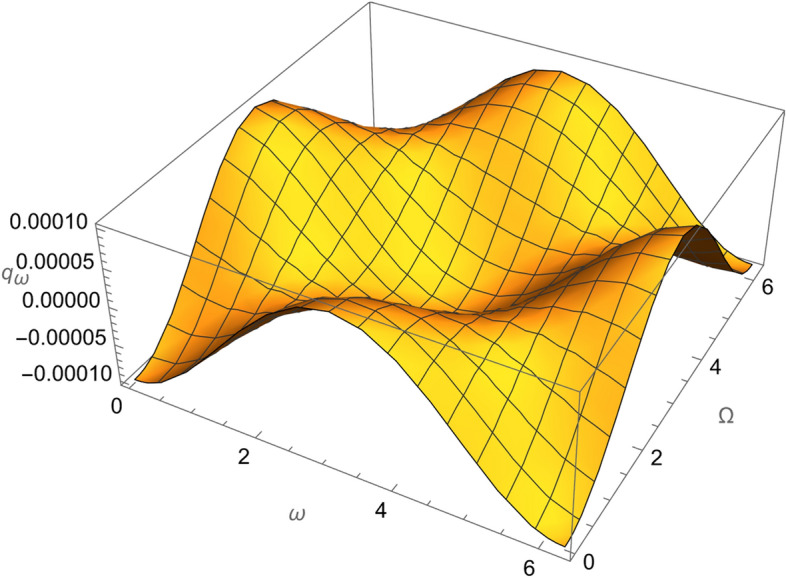
The variation of $\omega$ and $\Omega$ with $q_{\omega }$ for LAGEOS type satellite as a mid-Earth orbit.

**Figure 11:**
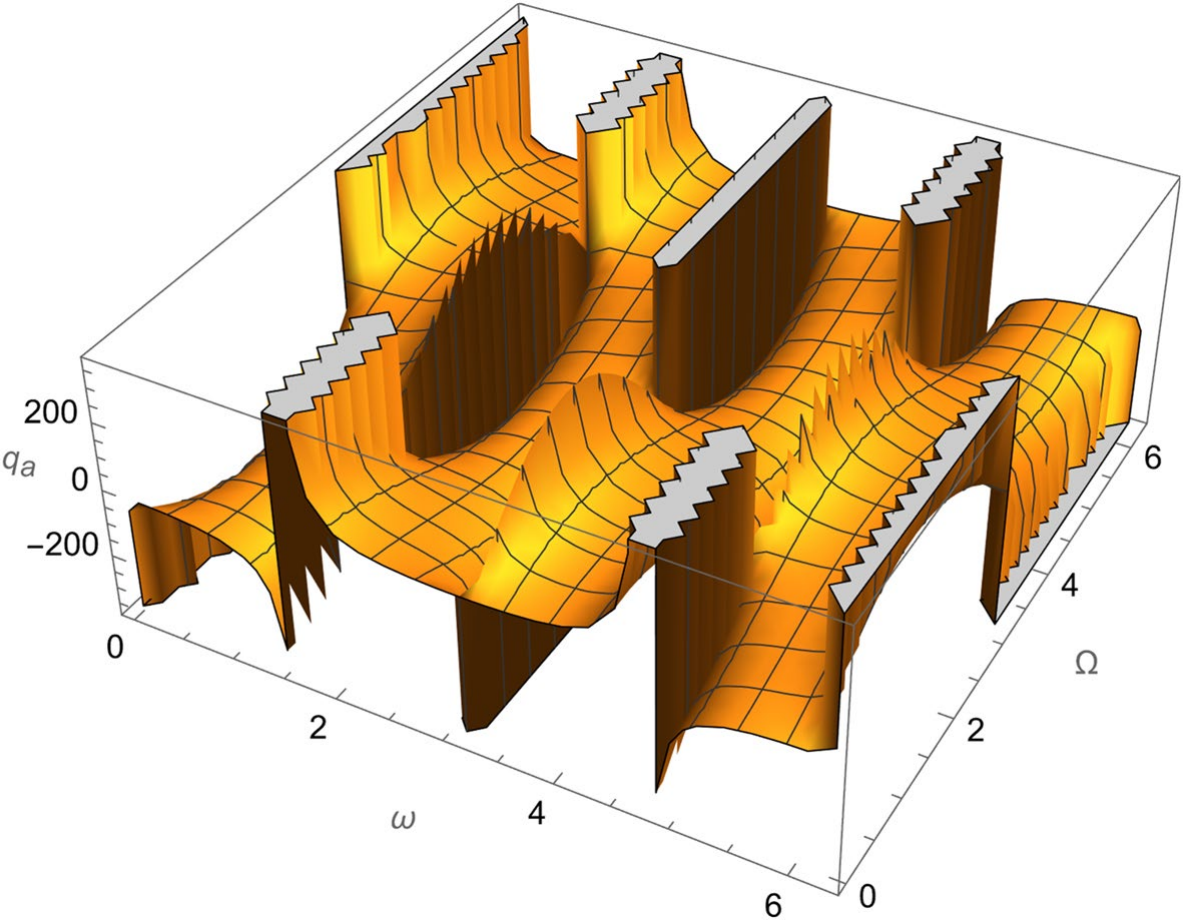
The variation of $\omega$ and $\Omega$ with $q_{a}$ for Geostationary type satellite as a high Earth orbit.

**Figure 12:**
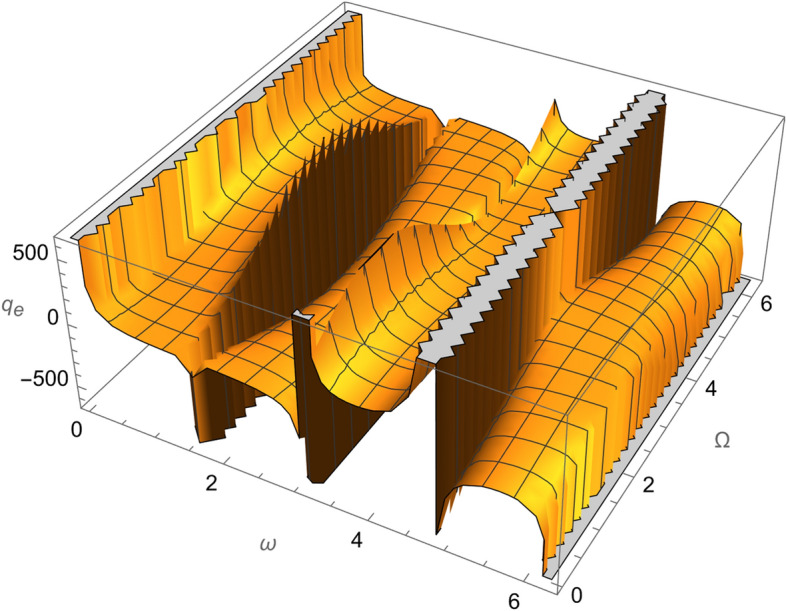
The variation of $\omega$ and $\Omega$ with $q_{e}$ for Geostationary type satellite as a high Earth orbit.

**Figure 13:**
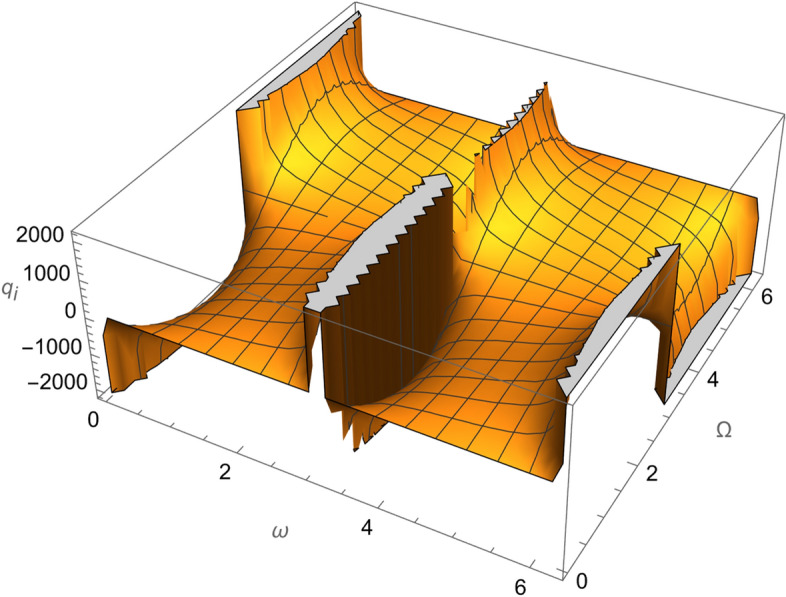
The variation of $\omega$ and $\Omega$ with $q_{i}$ for Geostationary type satellite as a high Earth orbit.

**Figure 14:**
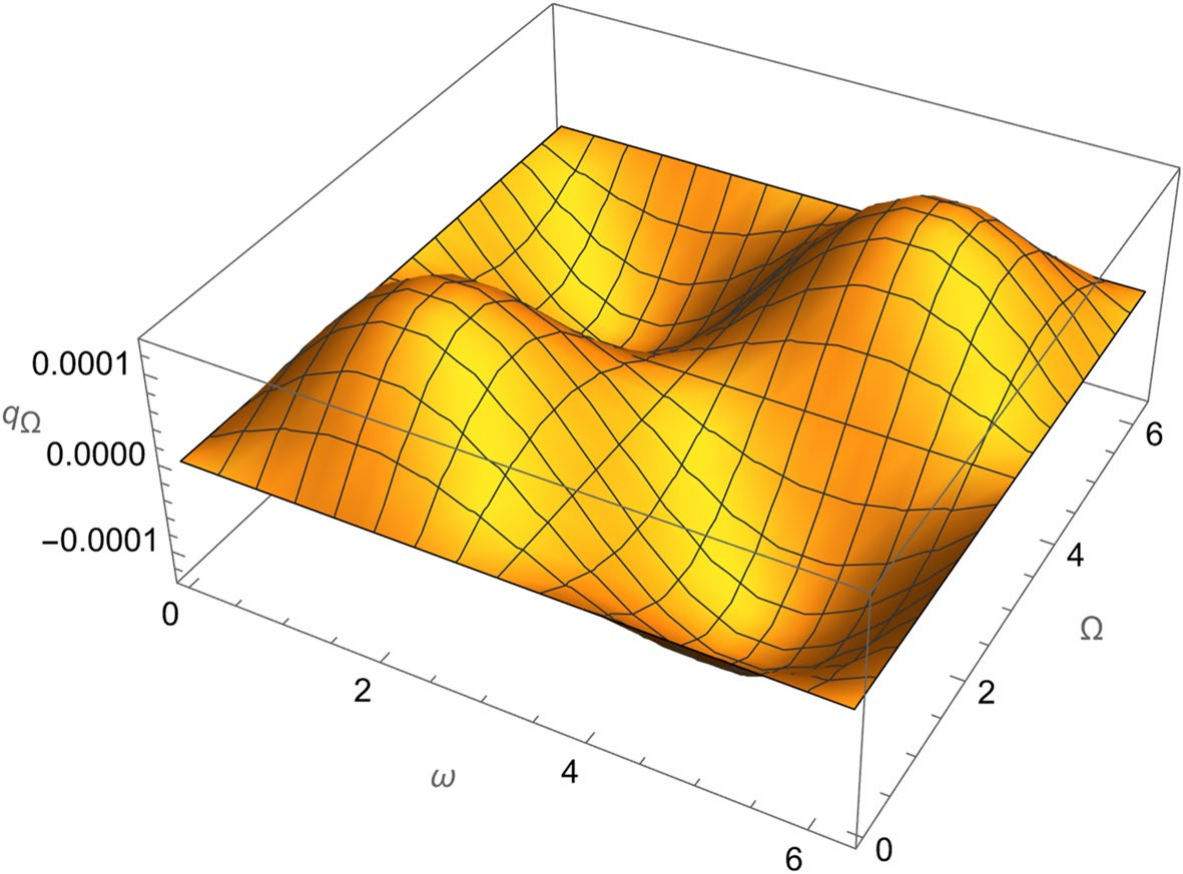
The variation of $\omega$ and $\Omega$ with $q_{\Omega }$ for Geostationary type satellite as a high Earth orbit.

**Figure 15:**
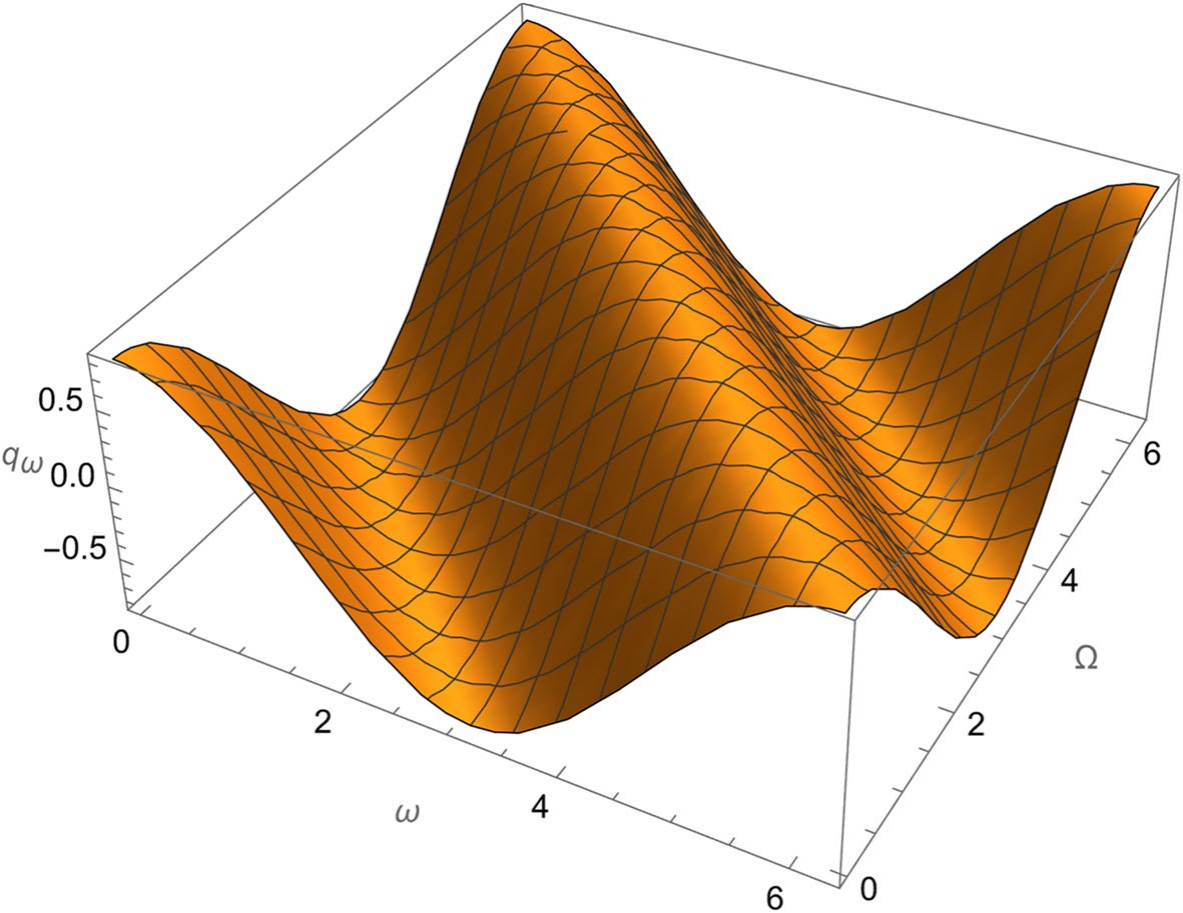
The variation of $\omega$ and $\Omega$ with $q_{\omega }$ for Geostationary type satellite as a high Earth orbit.

We conclude that Lorentz force can be used to balance perturbations of solar radiation pressure since they are of similar order for the applicable values of charge that can be produced with the present technology. But for example, it will be not applicable to balance air drag or earth’s oblateness with Lorentz force for low earth orbits. The work mainly studies how to get use of Lorentz force in the process of balancing. It could be used among set of forces to balance different set of perturbing forces. The technical part of this control can be handled by adding instrument onboard of spacecraft to measure the charge accumulated on the spacecraft’s surface, then special devices will generate the required charge value (magnitude and sign) according to the calculations of Eqs. (9.1)–(9.5) and according to the element we would like to balance.

## Data Availability

The datasets generated and analyzed during the current study are available in the^19^ (https://www.sciencedirect.com/science/article/abs/pii/S0094576512000732) and^20^ (https://ui.adsabs.harvard.edu/abs/1987npsg.book.....M).

## Abbreviations

LF: Lorentz force
LS: Lorentz spacecraft
SRP: Solar radiation pressure
LPE: Lagrange planetary equation
